# Chenodeoxycholic Acid Ameliorates AlCl_3_-Induced Alzheimer’s Disease Neurotoxicity and Cognitive Deterioration via Enhanced Insulin Signaling in Rats

**DOI:** 10.3390/molecules24101992

**Published:** 2019-05-24

**Authors:** Firas H. Bazzari, Dalaal M. Abdallah, Hanan S. El-Abhar

**Affiliations:** 1 Department of Pharmacology & Toxicology, Faculty of Pharmacy, Cairo University, Cairo 11562, Egypt; dalaal.abdallah@pharma.cu.edu.eg (D.M.A.); hanan.elabhar@pharma.cu.edu.eg (H.S.E.-A.); 2 Department of Pharmacology & Toxicology, Faculty of Pharmaceutical Sciences and Pharmaceutical Industries, Future University in Egypt, Cairo 11835, Egypt

**Keywords:** Alzheimer’s disease, insulin resistance, chenodeoxycholic acid, Aβ_42_, BACE1, IRS-1/Akt/GLUT4, CREB/BDNF, GLP-1, PPARγ

## Abstract

Insulin resistance is a major risk factor for Alzheimer’s disease (AD). Chenodeoxycholic acid (CDCA) and synthetic Farnesoid X receptor (FXR) ligands have shown promising outcomes in ameliorating insulin resistance associated with various medical conditions. This study aimed to investigate whether CDCA treatment has any potential in AD management through improving insulin signaling. Adult male Wistar rats were randomly allocated into three groups and treated for six consecutive weeks; control (vehicle), AD-model (AlCl_3_ 50 mg/kg/day i.p) and CDCA-treated group (AlCl_3_ + CDCA 90 mg/kg/day p.o from day 15). CDCA improved cognition as assessed by Morris Water Maze and Y-maze tests and preserved normal histological features. Moreover, CDCA lowered hippocampal beta-site amyloid precursor protein cleaving enzyme 1 (BACE1) and amyloid-beta 42 (Aβ_42_). Although no significant difference was observed in hippocampal insulin level, CDCA reduced insulin receptor substrate-1 phosphorylation at serine-307 (pSer307-IRS1), while increased protein kinase B (Akt) activation, glucose transporter type 4 (GLUT4), peroxisome proliferator-activated receptor gamma (PPARγ) and glucagon-like peptide-1 (GLP-1). Additionally, CDCA activated cAMP response element-binding protein (CREB) and enhanced brain-derived neurotrophic factor (BDNF). Ultimately, CDCA was able to improve insulin sensitivity in the hippocampi of AlCl_3_-treated rats, which highlights its potential in AD management.

## 1. Introduction

Alzheimer’s disease (AD) is the most common cause of dementia worldwide, accounting for 60–80% of all cases [1]. While the past two decades were dominated by the “amyloid cascade hypothesis”, the majority of the developed medicinal agents, specifically selective amyloid-beta (Aβ) targeting drugs, have, so far, failed to show any significance in AD management when evaluated in clinical trials [2]. Such findings have prompted the rise of other hypotheses to explain AD pathology and identify other potential targets for drug treatment. 

In several animal and clinical studies, insulin resistance was identified as a major risk factor for cognitive deterioration, dementia, neuronal death and brain atrophy [3]. Impaired insulin signaling, as well is linked to alterations in Aβ metabolism and tau hyperphosphorylation, besides neuronal demise, reduced synaptic plasticity and memory defects [4]. To close the vicious cycle, Aβ oligomers, on the other hand, also alter the function of insulin receptors resulting in a reduced responsiveness to insulin which further promotes insulin resistance [5]. To date, several medicinal agents have shown promising outcomes in AD management through enhancing insulin signaling, which all call attention to insulin resistance as a vital target for AD treatment [2]. 

Farnesoid X receptors (FXR) are nuclear bile acid receptors responsible for cholesterol homeostasis and are commonly expressed in the liver, intestine and kidneys [6,7]. FXR were also found to impact insulin signaling, wherein the work of Zhang et al. [8] FXR knockout mice exhibited glucose intolerance and developed signs of insulin resistance, while FXR activation was able to increase liver glycogen synthesis, glycogen content, insulin sensitivity and repressed gluconeogenic genes. In several other studies, FXR activation was observed to improve glucose metabolism, reverse insulin resistance and enhance insulin sensitivity, which in turn highlighted the potential of FXR agonists (i.e., bile acids and their synthetic derivatives) as therapies for metabolic syndrome, diabetes and obesity [9,10,11,12]. More recently, Huang et al. [13] identified functional FXR in the brain neurons, which opened the gate for further research to explore their roles in the central nervous system (CNS).

Chenodeoxycholic acid (CDCA) is a naturally occurring bile acid and a potent FXR activator that can effectively regulate the expression of FXR targets [13,14]. Multiple bile acids have been detected in the human brain and are speculated to be essential for mediating multiple signaling pathways; nevertheless, their exact functions are yet to be determined [15]. Moreover, low plasma levels of bile acids were reported in AD patients as well as healthy-elderly individuals [16,17]. In the comprehensive work of Ackerman and Gerhard [18], the authors overviewed numerous results of various cell line, animal and human studies that point out the potential efficacy of bile acids in the treatment of multiple neurodegenerative disorders and, so far, none have investigated CDCA as a candidate for AD. Furthermore, the molecular mechanisms by which bile acids exert neuroprotection are far from clear, which in turn hinder their clinical application. Therefore, such findings have prompted us to investigate the potential of CDCA in counteracting AD-associated insulin resistance as a strategy to slow down AD progression using an aluminum chloride (AlCl_3_) model in rats.

## 2. Results

### 2.1. CDCA Improves Learning, Spatial Working Memory and General Activity in AD Rat Model 

**Figure 1:**
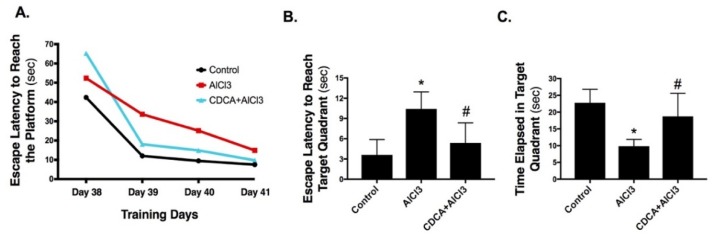

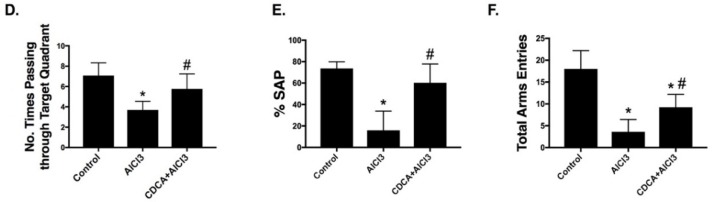
CDCA improves learning, spatial working memory and general activity in an AD rat model. (**A**) Morris Water Maze training, (**B**–**D**) probe test and (**E**,**F**) Y-Maze test. (**A**) Data represents means of trials (*n* = 13) per day for each group. (**B**–**F**) Data represents mean ± SD (*n* = 13). Statistical analysis was carried out using one-way ANOVA followed by Tukey’s *post-hoc* test. *p* < 0.05; as compared to (*) control and (#) AlCl_3_ group. AlCl_3_: aluminum chloride; CDCA: chenodeoxycholic acid; %SAP: spontaneous alteration percentage.

As shown in Figure 1, in the Morris Water Maze four training days AlCl_3_ increased (A) escape latency to reach the platform compared to the control group indicating the occurrence of learning defects. However, CDCA treatment was able to attenuate AlCl_3_ impact, effects that can be noted from the second day of the training course. In the probe test, AlCl_3_ elevated (B) escape latency to reach target quadrant (2.9-fold), while reduced both (C) time elapsed in target quadrant and (D) number of times passing through target quadrant by 56.8% and 47.9%, respectively when compared to the control. In contrast, CDCA treatment was able to hinder AlCl_3_-induced spatial memory defects and the measured values did not show any significant difference from those of the control group.

In the Y-maze test, Figure 1 shows a sharp decline in both (E) spontaneous alteration percentage (%SAP) and (F) total arms entries by 78.4% and 79.9%, respectively in AlCl_3_ group compared to the control group. On the other hand, CDCA treatment was able to reduce the negative impact of AlCl_3_ on rats’ spatial working memory and general activity, as indicated by the increased %SAP (3.8-fold) and total arms entries (2.6-fold) in comparison to the AlCl_3_ group.

### 2.2. CDCA Decreases Amyloid-Beta Production in AD Rat Model

**Figure 2:**
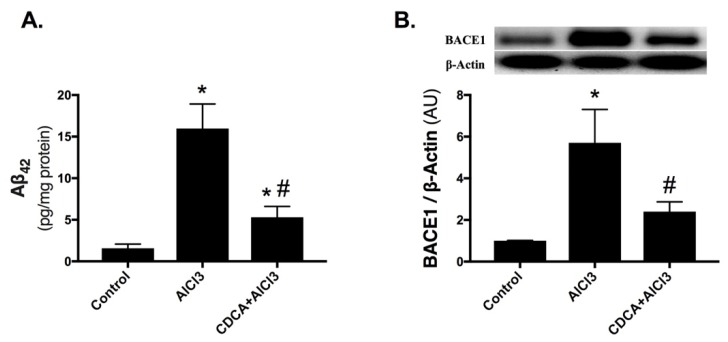
CDCA decreases amyloid-beta production in AD rat model. (**A**) Aβ_42_ level and (**B**) BACE1 expression. Data are presented as mean ± SD (*n* = 6 for Aβ_42_ and *n* = 4 for BACE1). Statistical analysis was carried out using one-way ANOVA followed by Tukey’s *post-hoc* test. *p* < 0.05; as compared to (*) control and (#) AlCl_3_ group. Aβ_42_: amyloid-beta 42; AlCl_3_: aluminum chloride; AU: arbitrary units; BACE1: beta-site amyloid precursor protein cleaving enzyme-1; CDCA: chenodeoxycholic acid.

As depicted in Figure 2, AlCl_3_ markedly elevated the hippocampal (A) amyloid-beta 42 (Aβ_42_) level (10.1-fold) and (B) beta-site amyloid precursor protein cleaving enzyme 1 (BACE1) expression (5.7-fold) compared to the control counterpart. On the contrary, CDCA treatment reduced Aβ_42_ level to reach 33.2% compared to AlCl_3_-only exposed animals and abated BACE1 protein expression to a value that was not significant from the control group.

### 2.3. CDCA Augments Hippocampal Insulin Signaling in AD Rat Model 

**Figure 3:**
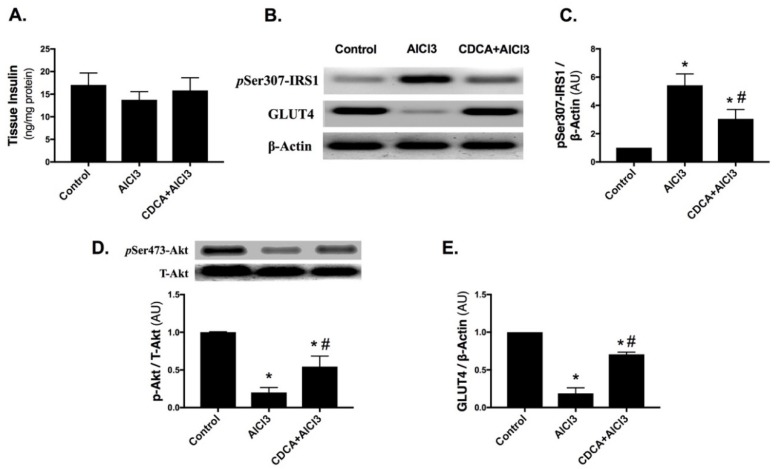
CDCA enhances (**A**–**E**) insulin signaling in AD rat model. Data are presented as mean ± SD (*n* = 6 for tissue insulin and *n* = 4 for western blot). Statistical analysis was carried out using one-way ANOVA followed by Tukey’s *post-hoc test*. *p* < 0.05; as compared to (*) control and (#) AlCl_3_ group. AlCl_3_: aluminum chloride; AU: arbitrary units; CDCA: chenodeoxycholic acid; GLUT4: glucose transporter type 4; pSer307-IRS1: phosphorylated insulin receptor substrate 1 at serine 307; pSer473-Akt: phosphorylated protein kinase B at serine 473; T-Akt: total protein kinase B.

In Figure 3, although no significant difference was observed in (A) tissue insulin among the three groups, the mean value in AlCl_3_-only treated group (13.8 ng/mg protein) was relatively lower compared to control (17.1 ng/mg protein) and CDCA-treated group (15.8 ng/mg protein). Nevertheless, AlCl_3_ has impaired insulin signaling, while treatment with CDCA enhanced it.

AlCl_3_ exposure boosted the protein expression of (B & C) phosphorylated insulin receptor substrate-1 at serine-307 residue (pSer307-IRS1) (5.4-fold), while declined the protein expression of (D) phosphorylated protein kinase B at serine 473 (pSer473-Akt), with the consequent depletion of (B & E) glucose transporter type 4 (GLUT4) in comparison to the control. However, CDCA treatment opposed the effects of the insult and lowered pSer307-IRS1 by 43.8%, while enhanced pSer473-Akt along with the overall p-Akt/T-Akt ratio (2.7-fold) and boosted GLUT4 (3.7-fold) as compared to the AlCl_3_-only treated group.

### 2.4. CDCA Improves Hippocampal GLP-1 and PPARγ Levels in AD Rat Model

**Figure 4:**
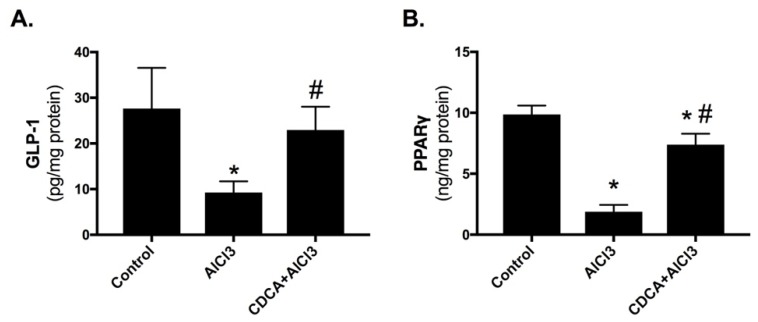
CDCA improves (**A**) GLP-1 and (**B**) PPARγ in AD rat model. Data represents mean ± SD (*n* = 6). Statistical analysis was carried out using one-way ANOVA followed by Tukey’s *post-hoc* test. *p* < 0.05; as compared to (*) control and (#) AlCl_3_ group. AlCl_3_: aluminum chloride; CDCA: chenodeoxycholic acid; GLP-1: glucagon-like peptide-1; PPARγ: peroxisome proliferator-activated receptor gamma.

As presented in Figure 4, (A) glucagon-like peptide-1 (GLP-1) and (B) peroxisome proliferator-activated receptor gamma (PPARγ) were markedly reduced by 67% and 81%, respectively in the AlCl_3_-only treated group compared to control. These effects were, however, opposed by the administration of CDCA, with GLP-1 value reaching about the normal range, while PPARγ had a considerable elevation (3.9-fold) compared to AD-model.

### 2.5. CDCA Promotes Hippocampal BDNF/CREB Pathway in AD Rat Model

**Figure 5:**
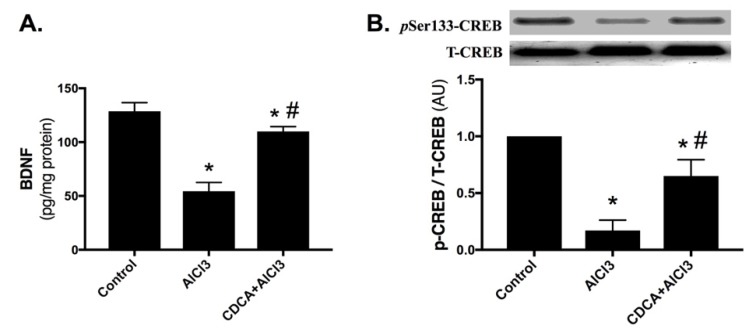
CDCA promotes hippocampal (**A**) BDNF level and (**B**) pSer133-CREB/T-CREB ratio in AD rat model. Data represents mean ± SD (*n* = 6 for BDNF and *n* = 4 for western blot). Statistical analysis was carried out using one-way ANOVA followed by Tukey’s *post-hoc* test. *p* < 0.05; as compared to (*) control and (#) AlCl_3_ group. AlCl_3_: aluminum chloride; AU: arbitrary units; BDNF: brain-derived neurotrophic factor; CDCA: chenodeoxycholic acid; pSer133-CREB: phosphorylated cAMP response element-binding protein at serine 133; T-CREB: total cAMP response element-binding protein.

In Figure 5, AlCl_3_ markedly lowered the hippocampal content of (A) brain-derived neurotrophic factor (BDNF) by 57.7%, and decreased the protein expression of (B) phosphorylated cAMP response element-binding protein at serine 133 (pSer133-CREB) and curtailed the p-CREB/T-CREB ratio by 83% when compared to the control group. Most importantly, CDCA treatment boosted BDNF content (2-fold) and augmented pSer133-CREB protein expression and the overall p-CREB/T-CREB ratio (3.8-fold), compared to AlCl_3_-only treated group.

### 2.6. CDCA Reduces AlCl_3_-Induced Neurodegeneration

**Figure 6:**
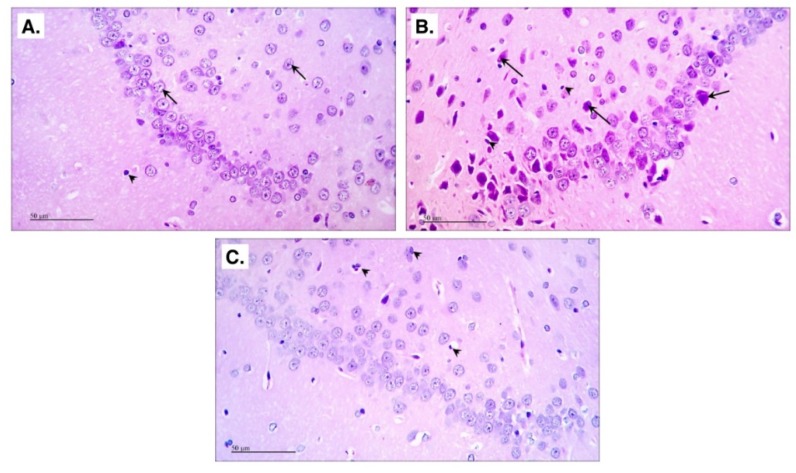
CDCA preserves normal histological features in AD rat model. The figure shows H&E photomicrographs of hippocampus CA1 region. (**A**) Control (arrows = intact neurons with vesicular nuclei; arrow-heads = glial cells), (**B**) AD-model group (arrows = degenerated neurons; arrow-heads = glial infiltration) and (**C**) CDCA-treated group (arrow-heads = glial infiltration). The figure scale bar = 50 μm. AlCl_3_: aluminum chloride; CA1: *Cornu Ammonis* 1; CDCA: chenodeoxycholic acid; H&E: Harris Hematoxylin and Eosin.

As depicted in Figure 6, (A) the control group shows normal histological structures of the hippocampus *Cornu Ammonis* 1 (CA1) region; however, exposure to (B) AlCl_3_ resulted in a severe neuronal degeneration with marked shrunken dark basophilic neurons in the polymorphic and pyramidal layers of CA1 region, as well as an increase in glial cells infiltration. 

Nonetheless, in (C) CDCA-treated group most of the neurons appear intact with mild glial infiltration.

## 3. Discussion

The current results demonstrate that CDCA successfully mitigated an early stage of AlCl_3_-induced cognitive impairments and delayed its progression. CDCA was able to improve rats’ learning, spatial working memory and general activity that were notably associated with reduced BACE1 to decrease Aβ_42_, a major AD hallmark. CDCA considerably acted via sextet players that converge to improve insulin sensitivity, enhance cognition and preserve normal histological features. CDCA activated the insulin pathway and increased the hippocampal contents of GLP-1, PPARγ, BDNF and CREB, besides abating Aβ_42_ to facilitate neuronal survival and halt AD symptoms.

Although the neuroprotective effects of several bile acids have been previously recited [18,19], yet the effect of CDCA was only detected in patients with cerebrotendinous xanthomatosis, a genetic disorder that has systemic and neurological manifestations along with the deficiency in CDCA [20]. These data, hence, have opened a new avenue for the potential usefulness of bile acids against CNS disorders. As shown in the current work, CDCA reduced AlCl_3_-induced cognitive defects as assessed by Morris Water Maze and Y-maze tests, these results were paralleled by a diminution in the measured AD markers; viz., BACE1 and Aβ_42_. The latter two parameters, however, were boosted in the AlCl_3_ treated animals which match earlier findings reporting the effect of AlCl_3_ on these markers [21,22] and spatial memory as well [23].

The suggested alterations in insulin and its receptor and their pathological relevance to sporadic AD date back to 1998 [24]. Furthermore, insulin signaling in the hippocampus and cerebral cortex has proven to be essential for memory formation, learning and synaptic plasticity [25]. These verities were verified in the present work, where CDCA activated insulin signaling, while it was blunted in the AlCl_3_-only treated group. Failure of IRS1 to turn on the insulin signaling pathway, due to serine phosphorylation, results in a decrease in the hippocampal contents of p-Akt and GLUT4, as shown in the current model. The effects of AlCl_3_ on these crucial molecules in the insulin trajectory have been narrated previously, as AlCl_3_ insult was found to reduce p-Akt/GSK-3β cue [26] as well as mRNA and protein content of skeletal GLUT4 upon studying the effects of AlCl_3_ exposure on glucose homeostasis [27]. However, the present work is the first to examine and verify the defects in insulin signaling in the hippocampi of AlCl_3_-treated rats to demonstrate that insulin resistance and wrecked insulin signaling have started when AlCl_3_ insult promoted IRS1 serine phosphorylation. In turn, the latter halted the phosphorylation (i.e., activation) of Akt and terminated the effect of its downstream molecule GLUT4. Moreover, the present results show that this mutilation was devoid of a significant change in hippocampal insulin level, which points out that insulin resistance depends on the whole pathway rather than the level of insulin alone.

By reducing the inactive pSer307-IRS1, CDCA was able to enhance pSer473-Akt and GLUT4; thus, CDCA-mediated insulin sensitivity can be a reason behind the improved cognitive function observed in the current investigation. These effects were previously recapitulated in a number of in-vitro studies, in which CDCA treatment was found to reduce pSer307-IRS1 and enhance insulin-sensitivity along with the release of anti-inflammatory adipokines in palmitate-treated 3T3-L1 cells and adipose tissues of rats fed high-fat diet [28,29].

Despite the scarcity of data about the role of CDCA in CNS, yet in the periphery, a 2016 study by Zhang et al. [30] reported that improved insulin sensitivity was achieved through CDCA agonistic activity to FXR. In addition, CDCA was found to upregulate the expression of GLUT4, which was suggested to be mediated through FXR binding to GLUT4-FXR response element (FXRE) in the GLUT4 promoter; thus, indicating that FXR is a new transcription factor for GLUT4 and highlighting the role of FXR and its agonists in insulin sensitivity and glucose homeostasis [31]. Furthermore, Shihabudeen and his colleagues [29] have stated that CDCA-improved insulin sensitivity was obtained via CDCA ability to promote the release of anti-inflammatory adipokines, yet without examining the insulin trajectory. To further prove the involvement of FXR in mediating insulin sensitivity, Renga et al. [32] have shown that the activation of FXR in βTC6-mice cells via 6E-CDCA, a semi-synthetic CDCA analog with more FXR affinity, increased Akt phosphorylation and GLUT translocation. Additionally, patients with type-2 diabetes and non-alcoholic fatty liver, who received 6E-CDCA treatment for 6 weeks, had a decrease in insulin resistance and inflammatory markers [33]. 

In AD, insulin resistance is mainly attributed to defects in insulin downstream post-binding actions that may develop in response to multiple factors, such as the accumulation of Aβ oligomers as well as other stress and inflammatory-related molecules [34,35,36]. To highlight the intimate cross-talk between AD markers and insulin resistance, earlier findings have illustrated that extracellular accumulation of Aβ oligomers activates an array of kinases to inactivate IRS response by promoting serine phosphorylation, namely Ser312 (in humans) and Ser307 (in rats) [4,37]. Therefore, IRS phosphorylation on these serine residues was considered as an indicator of insulin resistance that prevents the active insulin interaction with its receptors [37], a fact that supports the present effect of AlCl_3_ on heightening the hippocampal content of pSer307-IRS1. Consequent to inactive IRS, other kinases (e.g., GSK-3β) also take a part in the abnormal hyperphosphorylation of the intracellular tau proteins, which can be considered as a possible link between extra- and intra-cellular events found in AD [4,38]. Moreover, Aβ oligomers also broaden their effect and internalize insulin receptors [4]. Besides, insulin receptors were reported to be reduced through the binding of Aβ to the surface of the neuronal membrane, namely at synaptic clefts, leading eventually to insulin resistance and loss of synapse [39]. Thus, the ability of CDCA to abate BACE1 and Aβ_42_ can also be another key step in the activation of insulin signaling and improved cognition, which constructs the second arm of the present hexagon responsible for CDCA beneficial outcomes.

Apart from boosting insulin signaling, treatment with CDCA also enhanced the hippocampal level of GLP-1 to concur with the previous work by Nielsen et al. [40], where CDCA enhanced GLP-1 secretion in patients who underwent Roux-en-Y gastric bypass. Increased GLP-1 is the third player in the present sextet to explain improved insulin sensitivity and recovered cognition, as GLP-1 does not only play a key role in glucose metabolism but also facilitates numerous cognitive and other CNS-related functions [41]. Several studies have suggested that the enhanced GLP-1 secretion is achieved through the action of bile acids on their membrane type receptor, also known as Takeda G protein-coupled receptor 5 (TGR5) [40,42,43]. Nonetheless, CDCA is known to have a weak affinity to TRG5 [44,45], which suggests the involvement of other mechanisms to mediate this action. A possible explanation can be found in the recent work by Pathak et al. [46] which identified an “FXR-responsive element on the TGR5 gene promoter”. In other words, FXR activation induces TGR5 and both further cooperate to promote GLP-1 secretion.

Additionally, the fourth player in the insulin sensitivity orchestrate is the CDCA-mediated increase in the hippocampal content of PPARγ, as shown herein. This receptor is well known for its capacity to mediate insulin signaling via augmenting the release of adiponectin and the translocation of GLUT4 to the surface of skeletal myocytes and adipocytes [47,48]. Other studies have documented the cross-talk between FXR and PPARγ in alleviating liver fibrosis [49] and other metabolic diseases like nonalcoholic fatty liver disease [50]. Hence, these data may correlate to the present work and emphasize the possible involvement of these two receptors in mediating CDCA-related neuroprotection and insulin sensitivity, seen in our study.

Parallel to impaired insulin signaling, AlCl_3_ abated the hippocampal content of BDNF and its downstream molecule p-CREB. The impact of AlCl_3_ on BDNF concurs with previous data reporting on the low levels of BDNF in AD, which is considered as a marker of neurodegeneration and disease progression [51]. The occurrence of memory deficits as well as reduced levels of BDNF, CREB and IRS/PI3K/Akt have been observed in a hepatic cirrhosis-induced minimal hepatic encephalopathy model, which in turn insinuates that a positive overlap between BDNF and insulin signaling does exist [52]. On the other hand, Suwa et al. [53] have noted that BDNF promotes GLUT4 protein expression in the gastrocnemius muscle; accordingly, this neurotrophic factor might improve insulin sensitivity by binding to its tropomyosin receptor kinase B (TrkB) to activate Akt pathway, which provides an additional explanation for CDCA-mediated insulin sensitivity and forms the fifth side of our hexagon. Upon binding to its receptor (i.e., TrkB), BDNF activates IRS response that further activates its downstream signaling PI3K/Akt leading eventually to the enhancement of insulin actions [54]. To ensure the cross-talk between the current players, Kariharan et al. [55] have previously identified a novel signaling mechanism linking PPARγ and BDNF in the brain, where PPARγ was found to regulate BDNF promoter activity and ameliorate diabetes-associated cognitive impairment.

Apart from insulin signaling, increased Akt and the binding of BDNF to TrkB entail the activation of CREB, the last player in the insulin sensitivity sextet. In 2015, Xiang and coworkers [56] reported an impaired cognition as well as the downregulation of Akt, CREB and BDNF in the hippocampi of streptozotocin-treated rats, results that may offer a new way to impede diabetes-induced encephalopathy and cognitive deficits in AD. In a feed-forward loop, the last two players communicate, where activated/phosphorylated CREB is known to upregulate the expression of BDNF and other pro-survival genes [54,57]. This verity is documented herein, as CDCA treatment opposed the AlCl_3_ harm through activating CREB, besides enhancing BDNF and insulin signaling along with cognition. 

**Figure 7:**
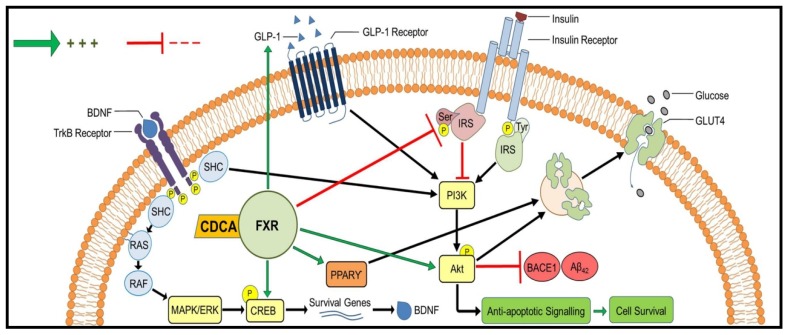
CDCA-mediated effects in ameliorating insulin resistance and AD pathologies. The vital role of CDCA in enhancing insulin signaling is mainly attributed to its ability to lower IRS1 serine phosphorylation, promote Akt activation and reduce Aβ production. CDCA also elevates hippocampal contents of GLP-1 and PPARγ that both take a part in insulin signaling. Moreover, CDCA enhances the activation of CREB to promote BDNF and neuronal survival. Akt: protein kinase B; Aβ_42_: amyloid-beta 42; BACE1: beta-site amyloid precursor protein cleaving enzyme 1; BDNF: brain-derived neurotrophic factor; CDCA: chenodeoxycholic acid; CREB: cAMP response element-binding protein; ERK: extracellular signal–regulated kinase; FXR: farnesoid X receptor ; GLP-1: glucagon-like peptide 1; GLUT4: glucose transporter type 4; IRS1: insulin receptor substrate 1; MAPK: mitogen-activated protein kinase; PI3K: phosphoinositide 3-kinase; PPARγ: peroxisome proliferator-activated receptor gamma; TrkB: tropomyosin receptor kinase B.

Collectively, CDCA-mediated effects in ameliorating insulin resistance and AD-associated pathologies were achieved through CDCA ability to modulate IRS-1/Akt/GLUT4, GLP-1/Akt/GLUT4, PPARγ/GLUT4, BDNF/CREB and BACE1/Aβ_42_, Figure 7.

## 4. Materials and Methods

### 4.1. Animals and Ethical Considerations 

Adult male Wistar rats obtained from The National Research Center (Cairo, Egypt) and weighing (230 ± 20 g) were used. Animals were kept under controlled conditions of room temperature (25 ± 2 °C), humidity (60 ± 10%) and alternating 12-h light/dark cycle, in the Experimental Animals Housing Unit facility in the Faculty of Pharmacy, Cairo University (Cairo, Egypt). Rats fed standard pellet diet (Al-Majd Co., Monufia, Egypt) and had free access to food and water *ad libitum*. Rats were allowed an acclimatization period of two-weeks prior to the start of drug treatment. 

Animal handling and all procedures were performed in accordance with and strictly adhered to the “Guide for The Care and Use of Laboratory Animals” 8th edition [58] adopted by and subjected to the approval of the “Research Ethics Committee” of the Faculty of Pharmacy, Cairo University (approval I.D. number: PT 2032). Extensive efforts were undertaken to reduce animal suffering.

### 4.2. Chemicals, Antibodies and Kits 

CDCA was purchased from Sigma-Aldrich (Darmstadt, Germany; CAT#: C9377); and both AlCl_3_ and sodium bicarbonate (NaHCO_3_) were purchased from Oxford Lab Chem (Mumbai, India).

Western blot antibody of pSer473-Akt was obtained from MyBioSource Inc. (San Diego, CA, USA; CAT#: MBS003071), whereas the remaining primary antibodies were obtained from ThermoFisher Scientific (Waltham, MA, USA); viz., anti-pSer307-IRS1 (CAT#: 44-813G), anti-T-Akt (CAT#: MA5-14916), anti-GLUT4 (CAT#: MA1-83191), anti-pSer133-CREB (CAT#: MA5-11192), anti-T-CREB (CAT#: MA1-083), anti-BACE1 (CAT#: MA1-177) and anti-β-Actin (CAT#: PA1-183). 

Sources for enzyme-linked immunosorbent assay (ELISA) kits are mentioned in brackets; Aβ_42_ (MyBioSource Inc.; CAT#: MBS726579), tissue insulin level (MyBioSource Inc.; CAT#: MBS724709), BDNF (RayBiotech, Peachtree Corners, GA, USA; CAT#: ELR-BDNF), GLP-1 (RayBiotech; CAT#: EIAR-GLP1) and PPARγ (CUSABIO, Houston, TX, USA; CAT#: CSB-E08624r).

### 4.3. Induction of Alzheimer’s Model and CDCA Dosing

AlCl_3_ is widely used as a model to mimic AD neurotoxicity [59]. To be consistent with the 3Rs (i.e., replacement, reduction and refinement) and the ARRIVE guidelines [60], a pilot study was conducted prior to the main work to test different dosing regimens of AlCl_3_ available in the previous literature. Based on the pilot outcomes, AlCl_3_ dose of 50 mg/kg/day (i.p) has resulted in a significant decline in rats’ cognition over a period of six weeks with the lowest mortality rate.

Currently, there is no clear and well-established reference dose of CDCA for in-vivo CNS application in rats. However, in humans, the CDCA dose of 15 mg/kg/day p.o was observed to ameliorate cognitive impairments associated with cerebrotendinous xanthomatosis [18,61]. Therefore, using the Food and Drug Administration (FDA) guide for dose conversion (i.e., Rat dose = Human dose × 6.2) [62], an estimate dose of (~90 mg/kg/day) CDCA was obtained. Furthermore, CDCA has a high oral bioavailability and mainly soluble in organic solvents, such as dimethyl sulfoxide and ethanol [63]. Nevertheless, to avoid the undesired effects of organic solvents on rats’ behavior [64,65], which may interfere with the study results, a CDCA suspension was prepared using 8.4% (1 mmol/mL) NaHCO_3_, as described in the European Medicines Agency leaflet of Chenodeoxycholic acid Leadiant^®^ [66].

### 4.4. Experimental Design

**Figure 8:**
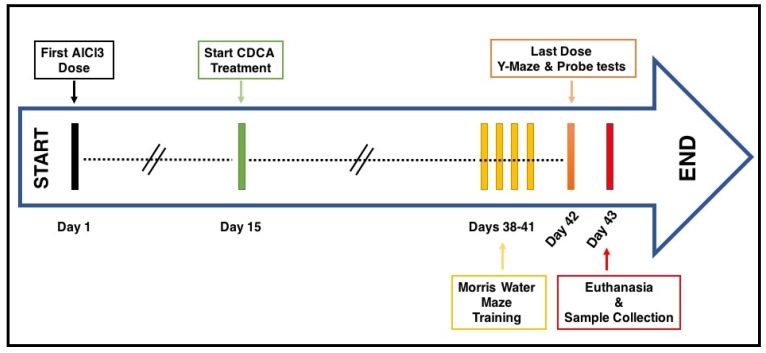
Schematic illustration of the experimental design. Rats were divided into three main groups; control, AlCl_3_ and CDCA-treated groups. The experiment lasted for 42 days (i.e., 6 weeks), in which AlCl_3_ treatment started on day 1 in the second and third group, while CDCA administration in the third group started on day 15. On days 38-41, rats were trained in the Morris Water Maze. On day 42, rats received the last treatment and the Y-maze and probe tests were performed. On day 43, rats were euthanized and samples were collected for further biochemical analysis and histopathological examination. AlCl_3_: aluminum chloride; CDCA: chenodeoxycholic acid.

A total of 39 rats were randomly divided into three groups (*n* = 13) and treated daily for six consecutive weeks as the following: (1) control group, in which animals received the drug vehicle (i.e., normal saline i.p and 8.4% NaHCO_3_ p.o from day 15), (2) AD-model group, in which rats were treated with AlCl_3_ (50 mg/kg/day i.p dissolved in normal saline) and 8.4% NaHCO_3_ p.o from day 15 and (3) CDCA-treated group, in which rats received AlCl_3_ (50 mg/kg/day i.p starting from day one) and CDCA (90 mg/kg/day p.o in 8.4% NaHCO_3_, starting from day 15), Figure 8. CDCA treatment in the third group was delayed to day 15 in order to allow AlCl_3_ to accumulate in the brain and induce mild/moderate toxicity and cognitive impairment (i.e., mimicking an early stage of AD), which was assessed in the pilot conducted prior to the main work, in addition to the available literature reporting on a disruption in the brain catecholamine content [67], an increase in CNS oxidative stress [68], histopathological damage in the brain tissues [69] and a decrease in total rats’ weights [70], after a short-term (7–15 days) treatment with AlCl_3_. 

Four days before the study’s end date (i.e., day 38, 39, 40 and 41) rats were trained in the Morris Water Maze. On the last day of the study (i.e., day 42), animals received the last treatment dose and the Y-maze and probe tests were later performed. After 24 h (i.e., on day 43), rats were euthanatized via rapid decapitation and the brains were harvested. For further biochemical analysis and histopathological examination, the samples were divided into three major subsets; (1) western blot (*n* = 4), (2) ELISA (*n* = 6) and (3) histopathological examination (*n* = 3).

### 4.5. Behavioral Testing 

#### 4.5.1. Morris Water Maze

Morris Water Maze protocols [71,72] were performed to assess rats’ spatial memory and learning. In this study, a round-shape water pool; 1.5 m in diameter, 0.6 m deep, 0.4 m water fill, (24 ± 2 °C) water temperature and placed in a soundproof room, was used. The pool was divided into four identical quadrants and a movable escape platform (9 cm in diameter) was fixed in the center of one quadrant throughout training days. On the training days, rats were allowed 12 training sessions (i.e., three sessions/day) of 2 min each. Escape latency, time needed for each rat to reach the platform, was visually recoded. Successful rats were allowed to stay on the platform for 10 s before they were removed; however, if the rat failed to reach the platform within 2 min, it was gently guided towards the platform and allowed to stay on it for additional 30 s. In the trials where rats failed to reach the platform, a time of 2 min was recorded as the trial time. On the 5th day, the platform was removed for the probe test (i.e., retrieval trail), in which each rat was allowed a total time of 1 min to explore the pool. In the probe test, escape latency (i.e., time to reach the target quadrant), time elapsed in the target quadrant and the number of times passing through the target quadrant were all visually recorded. After every trial, the rat was gently removed from the maze, dried and placed in a clean housing with available food and water *ad libitum*. All items in the room, including; clues, posters, etc., remained constant throughout the training and test days.

#### 4.5.2. Y-Maze: Spontaneous Alternation

Y-maze is a hippocampal-dependent spatial working memory test used to measure the willingness of rodents to explore new environments [73]. The test was used to obtain a clearer picture of the treatment outcomes and its effects via assessing spontaneous alterations. The Y-maze consists of three identically angled (120°) brown-colored wooden arms; 0.4 m in length, 0.12 m wide and 0.35 m high; and labeled A, B and C. The maze was placed in a soundproof room to prevent any external noise or interference that may influence the rats’ behavior. Animals were moved into the room 20 min prior the start of the test, then each rat was introduced to the maze and had a total time of 5 min to move freely inside it [74]; and the entry pattern of the arms was visually recorded. The arm entry was counted if all the rat’s hind paws were placed inside the arm. The maze arms were thoroughly cleaned between sessions in order to remove any remaining odors or residues. An alteration is defined as “successive entries into the three arms on overlapping triplet set” (e.g., ABC, CBA, BCA etc.) [21]. The recorded number of alterations and the total arms entries were used to calculate %SAP via the following equation [21]:

$$\%SAP=\frac{number\;of\;alternations}{total\;arms\;entries-2}\times 100$$

### 4.6. Tissue Sampling

For the estimation of the biochemical parameters, the harvested brains (*n* = 10) were rapidly dissected and the hippocampi were collected and stored in a (−80 °C) freezer. The hippocampi were homogenised in radio-immunoprecipitation assay (RIPA) buffer (150 mMNaCl, 0.1% Triton X-100, 0.5% sodium deoxycholate, 0.1% sodium dodecyl sulphate and 50 mM Tris-HCl pH 8.0) obtained from Bio Basic Inc. (Markham, ON, Canada; CAT#: PL005-5X10ML) to prepare samples for western blot and ELISA analysis. For histopathological examination, whole brains (*n* = 3) were flushed and fixed in 10% neutral buffered formalin (Sigma-Aldrich, St. Louis, MO, USA; CAT#: HT501128).

### 4.7. Biochemical Analysis

For western blot, after homogenization of the hippocampi in RIPA buffer, the protein content was quantified using Bio-Rad protein assay kit (Bio-Rad, Hercules, CA, USA). Thereafter, protein aliquots were isolated by sodium dodecyl sulfate–polyacrylamide gel electrophoresis (SDS-PAGE) and transferred to polyvinylidene difluoride (PVDF) membranes and blocked with 5% bovine serum albumin. The membranes were incubated with the primary antibodies against; viz. pSer307-IRS1, pSer473-Akt, T-Akt, GLUT4, pSer133-CREB, T-CREB and BACE1, overnight at (4 °C). Then, the membranes were probed with horseradish peroxidase (HRP)-conjugated secondary antibodies (Cell Signaling Technology, Danvers, MA, USA). The blots were identified through enhanced chemiluminescence (ECL) using Pierce™ ECL plus Western blotting substrate (ThermoFisher Scientific, Waltham, MA, USA; CAT#: 32132) performed according to the manufacturer’s guide. Ultimately, the amount of protein was quantified via calibrated laser imaging densitometer GS-800™ (Bio-Rad). The results were expressed as arbitrary units (AU). 

Other hippocampal biomarkers, including, Aβ_42_, tissue insulin level, BDNF, GLP-1 and PPARγ were measured via ELISA kits; and all procedures adhered to the instructions provided by the manufacturer. Parameters were normalized to the protein content measured according to Bradford (1976) protein assay protocol [75].

### 4.8. Histopathological Examination

Whole brains (*n* = 3) were flushed and fixed in 10% neutral buffered formalin for 72 h. Samples were trimmed, processed, dehydrated by serial grades of alcohol, cleared in xylene, infiltrated by synthetic wax and blocked out into Paraplast tissue embedding media. Sections (5 µm thickness) were cut by a rotatory microtome and stained with Harris Hematoxylin and Eosin (H&E), as a general examination staining method. Techniques were conducted as outlined by Bancroft and Layton [76]. Slides analysis and micrographs were taken using Leica Application Suite attached to Full HD microscopic imaging system (Leica Microsystems GmbH, Wetzlar, Germany). Preparation techniques and assessment were conducted by an external professional observer who was blinded throughout the whole process in order to avoid any sort of bias.

### 4.9. Statistical Analysis 

Data values are expressed as mean ± SD. One-way ANOVA was used to compare the difference between groups followed by Tukey’s as a *post-hoc* test to assess the significance among the groups’ means. GraphPad Prism 7.0c (GraphPad Software Inc., San Diego, CA, USA) software was used to carry out the statistical analysis and *p* < 0.05 was set as the minimal level of significance.

## 5. Conclusions

In conclusion, treatment with CDCA, through orchestrating six players, was able to ameliorate insulin resistance and enhance insulin sensitivity to improve cognition and reduce neurodegeneration in AlCl_3_-treated rats. The sextet players that were modulated by CDCA are BACE1/Aβ_42_, IRS-1/Akt/GLUT4, GLP-1/Akt/GLUT4, PPARγ/GLUT4, BDNF and CREB. At last, the study findings add to the piece of evidence that supports the use of bile acids as potential disease-modifying therapies for AD among other neurodegenerative disorders.
